# Effect of erbium yttrium aluminium garnet laser dentin conditioning on dental pulp stem cells viability

**DOI:** 10.1016/j.heliyon.2024.e26954

**Published:** 2024-02-29

**Authors:** Aryan Jafari, Mehdi Vatanpour, Nooshin Barikrow, Pouyan Razavi, Sohrab Tour Savadkouhi

**Affiliations:** a Dental School, Islamic Azad University of Medical Sciences, Tehran, Iran; b Endodontic Department, Dental School, Islamic Azad University of Medical Sciences, Tehran, Iran; c Department of Molecular and Cellular Sciences, Faculty of Advanced Sciences & Technology, Tehran Medical Sciences Branch, Islamic Azad University, Tehran, Iran; d Dental Material Research Center, Dental School, Islamic Azad University of Medical Sciences, Tehran, Iran

**Keywords:** Regenerative endodontics, Erbium YAG laser, EDTA, Dentin, Mesenchymal stem cells

## Abstract

**Objective:**

This study aims to investigate the effect of dentin conditioning by subablative Er:YAG (erbium-doped yttrium aluminium garnet) laser on dental pulp stem cells (DPSCs) viability.

**Methods:**

For this in-vitro experimental study, root fragments were longitudinally hemisected after decoronation of single-rooted extracted teeth and preparation of root canals. Prepared samples were randomly assigned to 2 experimental groups (n = 17) as follows; 1) laser conditioning: irradiation with Er:YAG laser beams (2940 nm, 50 mJ per pulse, 20 Hz) 2) Chemical conditioning: 1.5% NaOCl, followed by phosphate-buffered saline (PBS), 17% EDTA, followed by PBS as a final rinse. The samples were ultraviolet-sterilized, and DPSCs were seeded on the samples. MTT assay was performed after 1, 4 and 7 days of incubation to assess the cell viability (n = 5/group per day). Also, after 7 days, two samples of each group underwent SEM (scanning electron microscope) analysis. Statistical analysis was done using independent *t*-test, one-way ANOVA and two-way ANOVA at a significance level of 0.05.

**Results:**

Laser irradiated samples exhibited significantly higher cell viability of DPSCs on days 4 (p < 0.0001) and 7 (p < 0.0001), unlike day 1 (p = 0.131). SEM photomicrographs revealed that Er:YAG laser performed much better smear layer removal and created surface irregularities. Several different cell morphologies were observable on the laser-treated samples, which cells with cytoplasmic extensions being the most frequent.

**Conclusions:**

Dentin conditioning by Er:YAG laser enhances DPSCs viability and can be a valuable modality for conditioning dentin to perform regenerative endodontic procedures. Further clinical studies are suggested.

## Highlights

• Dentin samples were conditioned using Er:YAG laser or chemicals.
• Dental Pulp Stem Cells viability of laser group was significantly higher than chemically conditioned group.
• Laser-irradiated samples exhibited almost complete smear layer removal and clear dentinal tubules orifices.
• Flat cells with cytoplasmic extensions and lamellipodia seem to be more common in laser conditioned group.
• Round cells were the dominant morphological type in the chemical conditioned samples.

## 1 Introduction

Regenerative endodontic procedures (REPs) are now regarded as an effective therapy for immature permanent teeth with pulp necrosis. Successful REP results in root development, enhanced dentinal wall thickness, and apical closure. Furthermore, REPs have the potential to be used in adult teeth, such as extensive external root resorption and root perforation, according to a growing body of evidence [1]. To restore the pulp-dentin complex, mesenchymal stem cells, such as dental pulp stem cells (DPSCs) and their subpopulations, have been commonly employed [2].

Growth factors are bioactive molecules that have been embedded in the dentin matrix. These substances have a stimulating effect on stem cell development and growth. Dentin conditioning is a technique for releasing these components as well as preparing a treated surface for cell attachment, which is usually accomplished with the use of a chelating agent like EDTA [3]. The 17% EDTA solution was chosen for conditioning due to its capacity to demineralize the superficial dentin layer, expose growth factors stored in the dentin matrix, and remove the loosely adhered smear layer [4] and also decrease the bacterial endotoxins in the root canal space [5]. The smear layer which contains dentin debris and microorganisms, prevents stem cells from adhering to the dentinal walls, causing REPs' failure [6]. Organic components in the surface dentin layer, like collagen are exposed by EDTA treatment and play a critical part in cell adhesion [7]. However, since EDTA may impair cell function and blood clot formation, it may have an adverse impact on cell attachment [8].

Lasers have recently been shown to be efficient in teeth conditioning for cell and blood clot adhesion [9]. Er:YAG (erbium-doped yttrium aluminium garnet) laser is one of the most promising lasers in this field that can be used for conditioning the root canal as well as smear layer removal, which can result in a appropriate surface for fibrin clot development and cell adhesion [10,11]. However, the effect of Er:YAG conditioning on the viability of DPSCs in REPs is still unclear.

Therefore, this study aims to assess the impact of Er:YAG dentin conditioning of root canal dentinal walls on DPSCs viability. This study's results may prove helpful in the future of regenerative endodontics protocols.

## 2 Methods and materials

### 2.1 Specimen preparation

This experimental in-vitro study was conducted on 34 freshly extracted single-rooted human teeth with completely developed apices, extracted due to periodontal problems. Teeth with caries, fillings, calculus, fractures, root canal treatment and hypoplastic defects were excluded. Also, teeth were radiographically examined to ensure single canal presence and absence of resorptions and calcifications. The periodontal ligaments were scraped with a scalpel blade and periodontal curette from the root surface, and the teeth were maintained in 0.9% sterile saline till experiment time. The Tehran Islamic Azad University of medical sciences research ethics committee approved this study (IR.IAU.DENTAL.REC.1399.295).

The teeth were decoronated with a high-speed bur to obtain a 12 mm root length. The working length was determined by inserting an ISO #10 K-file (Dentsply Maillefer, Ballaigues, Switzerland) until its tip was visible beneath the apex and subtracting 0.5 mm from that length. Instrumentation was carried out using Neoniti files (Neolix, Evron, France) up to #25/8% size to the working length. During the filing process, the root canal was irrigated with 10 mL of 5.25% NaOCl. On the outer root surfaces of teeth, two vertical grooves were cut, taking care not to perforate the canal. A sterile stainless steel chisel was placed into one of the grooves and then twisted gently to split the root into two sections; each was randomly allocated into an experimental group. Root sections were dried for 24 h at room temperature before surface treatments.

### 2.2 Experimental design

The specimen was assigned to 2 groups (n = 17/group).

1) Laser conditioning: treated with Er:YAG laser (Fotona, Ljubljana, Slovenia) irradiation
2) Chemical conditioning: conditioned using NaOCl (Morvabon, Tehran, Iran), EDTA (Morvabon, Tehran, Iran) and PBS (phosphate-buffered saline).

Two samples from each group were separately seeded for SEM analysis on the 7th day.

### 2.3 Dentin surface treatment

Dentin conditioning for each group was carried out as follows;

Laser conditioning: irradiation with Er:YAG laser beams (2940 nm, 50 mJ, 20 Hz, super short pulse mode, air/water:6/6, 15 s with scanning movements, 7–8 mm perpendicularly) using non-contact tip less 90°-angled H02–N hand-piece.

Chemical conditioning: “1 ml of 1.5% NaOCl for 5 min, followed by 3 ml of PBS for 3 min (1 ml/min), 17% EDTA for 5 min, followed by 3 ml of PBS for 3 min (1 ml/min) as a final rinse” [12].

### 2.4 Viability assay

After ultraviolet sterilization, dentin sections were left to dry properly after all irrigation treatments and then put on 24 well-plates for seeding the cells with a culture medium. The human dental pulp stem cells (DPS-13, IBRC C10896) were obtained from the Iranian Biological Resource Center (Tehran, Iran) while cultured in Dulbecco modified Eagle medium (DMEM; BIO-IDEA, Tehran, Iran) -F12 (1:1) medium supplemented with 20% Fetal Bovine Serum, 2 mM l-Glutamine, in 37 °C at 5% CO_2_ atmosphere. From the 3rd passage, 1 × 10^4^ cells per sample were seeded on treated dentin specimens in 24-well plates, followed by incubation for 3 h at 37 °C to facilitate cell attachment. Every day the cultured medium was refreshed to maximize cell viability.

Cell viability was assessed by MTT assay. On days 1, 4, and 7, cells were harvested and transferred to separate 96 well-plates for each period. Viability was measured by adding 20 μl (0.5 mg/ml) of MTT solution and incubating at 37 °C for 4 h. The supernatants were collected, and 150 μl of dimethyl sulphoxide was added to each well. The samples' absorbance was measured at 570 nm in a microplate reader (SPECTROstar Nano, BMG LABTECH, Offenburg, Germany).

### 2.5 SEM

Two samples from each group on day 7 were chosen for scanning electron microscope (SEM) observation to evaluate the cell morphology and surface topography. Samples were rinsed twice in PBS at 37° and then fixed for 24 h in a 2.5% glutaraldehyde. The samples were then dehydrated for 10 min each in a series of ethanol (70, 85, 95, and 100% ethanol), then freeze-dried to a critical point. The samples were coated with gold and examined with an SEM (MIRA 3, TESCAN, Brno, Czech Republic) at a 15 kV accelerating voltage x5k and ×10 k magnifications. Photomicrographs were saved digitally in TIFF format.

### 2.6 Statistical analysis

Shapiro-Wilk test confirmed the normality of the data. Therefore, independent *t*-test, two-way ANOVA and one-way ANOVA with Tukey test as post-hoc analysis were employed for data analysis at significance level of 0.05. Statistical evaluation was performed by using SPSS 16 package (SPSS v.16, IBM, Chicago, IL., USA).

## 3 Results

### 3.1 Cell viability assay

The viability of DPSCs showed that the independent variables (conditioning group and time) were significant (p < 0.001) as well as the interaction (conditioning group versus time) (p < 0.001). DPSCs viability increased significantly over time in the laser conditioned group (p < 0.0001) and chemical conditioned group (p < 0.0001). DPSCs cell viability of laser conditioned group was significantly higher than chemically conditioned group (p < 0.001). The laser-treated group's cell viability was higher at all three observation periods; however, the difference was not significant on day 1 (p = 0.131), unlike days 4 (p < 0.0001) and 7 (p < 0.0001) **(**Table 1**).**

**Table 1:** Comparison of dental pulp stem cells’ viability of treatment groups in different periods (mean ± SD).

	1st day	4th day	7th day	P value
**Laser conditioning**	0.26 ± 0.09	1.04 ± 0.43	1.06 ± 0.19	<**0.0001**
**Chemical conditioning**	0.18 ± 0.06	0.33 ± 0.09	0.66 ± 0.12	<**0.0001**
**P value**	**0.131**	<**0.0001**	<**0.0001**	

There was a significant difference seen between the findings obtained on days 1 and 4 (p < 0.011), 1 and 7 (p < 0.001), and 4 and 7 (p < 0.015) in the EDTA group. Similarly, a significant difference was observed between the results obtained on days 1 and 4 (p < 0.0001), and 1 and 7 (p < 0.0001) in the erbium group. However, the difference between the results obtained on days 4 and 7 in the erbium group was not found to be significant (p = 0.890).

### 3.2 SEM observations

**Fig. 1:**
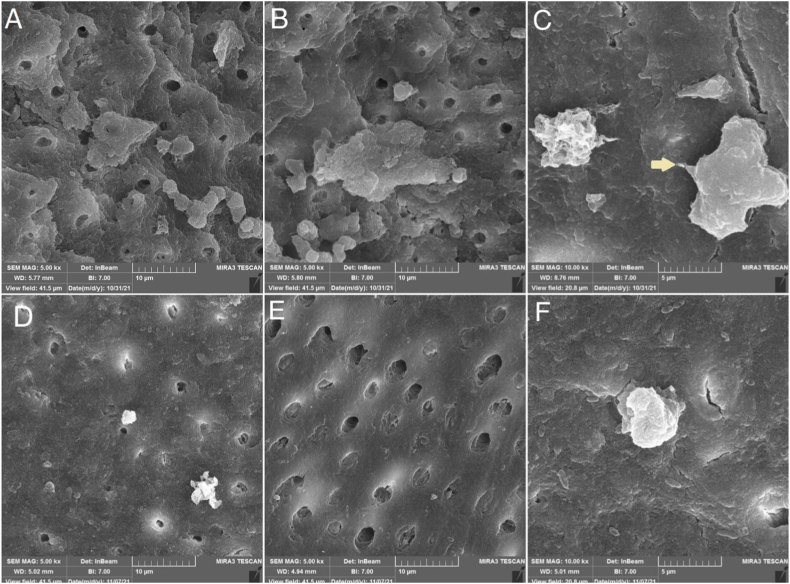
Scanning electron photomicrographs of dentinal surfaces treated with Er:YAG (A,B,C) or EDTA (D,E,F) and attached dental pulp stem cells 7 days after seeding. A, B) Er:YAG ablation pattern, open dentinal tubules, absence of smear layer & higher number of attached cells with various morphology patterns compared with EDTA group is evident. Note the absence of microcracks and thermal ablations. C) Stem cells' lamellipodia have facilitated their adhesion to the surface. D, E) Lower number of attached cells and presence of smear layer blocking dentinal tubules orifices are evident. F) A round cell attached to the surface without cytoplasmic extensions.

As shown in Fig. 1, laser-irradiated samples exhibited almost complete smear layer removal and clear dentinal tubules orifices, whereas there was some smear layer present on the dentinal tubules orifices in the conventionally treated group. The Er:YAG irradiated dentin samples exhibited uneven surfaces. Various cell morphologies were observed in the laser-conditioned group, as flat cells with cytoplasmic extensions and lamellipodia seem to be more common. Whilst, round cells were the dominant morphological type in the chemical conditioned samples.

## 4 Discussion

This experimental in-vitro study showed DPSCs cell viability on radicular dentin of laser-conditioned group was significantly higher than chemically (EDTA) conditioned group so the null hypothesis was rejected.

DPSCs, which have been employed in our study, contribute substantially to dentin regeneration and even complete pulp regeneration due to their promising neurogenic, angiogenic, and odontoblastic differentiation potential [13].

The ideal NaOCl concentration indicated for root canal therapy operations varies widely in the literature, ranging from 0.5 to 5.25%, which may prove to be toxic for the stem cells [14]. The 1.5% NaOCl was chosen as irrigant because of its robust antibacterial activity and capacity to preserve the survival of stem cells, which, as previously stated, play a critical role in REPs success [15].

EDTA application increases the expression of factors inducing stem cell differentiation, which is critical for forming new pulp tissues and mitigating the cytotoxic effects of NaOCl and intracanal medicaments [16]. A recent systematic review stated that EDTA conditioning has valuable advantages on REP. It promotes tissue neo-formation and speeds up the healing process by increasing dentin transforming growth factor-1 release and improving stem cell migration and differentiation [17]. Conversely, some research found that EDTA-treated dentin did not affect cell viability [18]. Also, others found that this conditioning had detrimental impacts on cell behavior, such as reduced stem cell survival and migration, and may have deleterious effects on tissue regeneration [12,19]. We followed the protocol suggested by Aksel et al. which emphasized the importance of final irrigation with PBS to remove any leftover EDTA from the dentin surface and showed the deleterious impact endodontic irrigants may have on cell activity. However, in our trial, that protocol failed to provide a suitable surface for cell attachment as the scattered cells maintained their round shape, unlike Aksel et al.'s SEM results [12].

Applying Er:YAG laser to tooth hard components is safe and does not harm the surrounding regions [20]. Because the wavelength of Er:YAG laser is well absorbed by water, a quick evaporation of water would result in the removal of hydroxyapatite crystals and the exposure of collagenous fibers at a temperature below their melting point [21]. As a conditioning agent, the Er:YAG laser has enhanced periodontal ligament cell adhesion to periodontitis-affected root surfaces [22]. Even at low energy levels, the Er:YAG laser is known to have considerable bactericidal effects, which makes Er:YAG laser irradiation a promising choice for REPs owing to the importance of proper non-toxic disinfection in REPs.

Excessive power may result in a superficial layer of mineral melt and cavitation flaws that hinder cell adhesion [23]. According to Feist et al. surfaces irradiated with 60 mJ/pulse Er:YAG laser had quicker cell adherence and proliferation than surfaces irradiated with 100 mJ/pulse Er:YAG laser [24]. As a result, determining the ideal Er:YAG laser irradiation settings for better cell attachment to root surfaces is critical [23,25]. Choosing the lowest energy level when utilizing an Er:YAG laser with subablative parameters is critical since higher energies generate microcracks, weaken the remaining dentinal root walls, and injure the peripheral and underlying tissues [26]. Thus, we used subablative Er:YAG laser settings to prevent surface melting and further structural weakening of immature dentinal walls which is crucial for REPs.

Smear layer removal during REPs is recommended as it can harbor bacteria that interfere with regeneration [27]. Based on our SEM observations, Er:YAG laser irradiation showed to be successful in smear layer removal and exhibited clean, scaly-like, coarse,a wider collagen fiber exposed area and uneven surface with open dentinal tubules without causing fractures or meltings, confirming Naghsh et al. and Cekici et al. results [10,28]. Though, some studies beg to differ as a study conducted by Kayonlcuoǧlu and Demiryürek suggested that Er:YAG laser (1.8 W, 120 mJ, 15 Hz, 381 J/cm^2^) was ineffective in removing the root canal smear layer and was not statistically different with the group irrigated with 5.25% NaOCl [29].

The surface texture of dentin seemed to influence DPSCs behavior and offer a surface for mechanical attachment and cellular growth in the Er:YAG irradiated samples. The lased surfaces' morphological roughness increased the adherence quality and quantity of DPSCs, which coincides with other studies' findings [11,23,25]. Under SEM, the substantial rate of cell attachment in Er:YAG treated samples corresponded to a noteworthy cell viability rate in MTT findings for this group.

The contact area of cells in cell morphology can be used as a predictor of cell affinity. Flat cells usually are firmly connected to the surface with their elongated cell bodies and lamellipodia, whereas round cells might be regarded weakly attached [30]. The SEM observations of Er:YAG treated samples demonstrated elongated cell bodies with extended cytoplasmic processes, possibly associated with higher cell viability in Er:YAG group.

A crucial step in cell culturing for in-vitro studies is preparing a sterile surface for seeding the cells. There have been some concerns and controversies regarding autoclave sterilization of the samples for in-vitro cellular studies. Sterilizing dentin before planting human cells with autoclave has been employed in some earlier studies [11,31]. On the other hand, Sulkala et al. claimed autoclave sterilization reduces dentinal collagenase activity but does not impact dentinal gelatinase activity [32]. Furthermore, autoclave sterilization may potentially harm endogenous growth factors in radicular dentin. Hence, the root sections were UV-irradiated at room temperature to maintain the bioactivity of growth factors embedded in the root canal wall in our experiments.

We used a non-tip Er:YAG laser in this in-vitro study which is not practicable in real clinical situations. When employing a laser to condition the dentinal walls, the laser should be directed toward the lateral root canal wall rather than the apex, as Stabholz et al. previously developed side-firing tips [33]. Side-firing tips are not currently available in the market for this purpose. Hence, manufacturers should be encouraged to fabricate side-firing tips.

## 5 Conclusions

Application of Er:YAG laser for dentin conditioning in REPs had promising results in this in-vitro study. Development of side-firing laser tips followed by further in-vitro and clinical studies using different Er:YAG laser settings may prove beneficial to the future of regenerative endodontics.

## Data availability statement

Data acquired from this study and utilized for statistical analysis can be obtained from the corresponding author upon reasonable requests.

## CRediT authorship contribution statement

**Aryan Jafari:** Writing – original draft, Data curation. **Mehdi Vatanpour:** Validation, Formal analysis. **Nooshin Barikrow:** Methodology, Investigation. **Pouyan Razavi:** Writing – review & editing, Software, Resources. **Sohrab Tour Savadkouhi:** Writing – review & editing, Supervision, Resources, Project administration, Conceptualization.

## Declaration of competing interest

The authors declare that they have no known competing financial interests or personal relationships that could have appeared to influence the work reported in this paper.
